# Electrophysiologic and molecular characteristics of cardiomyocytes after heavy ion irradiation in the frame of the ESA IBER-10 program

**DOI:** 10.1093/jrr/rrt163

**Published:** 2014-03

**Authors:** Johannes Frieß, Anja Heselich, Sylvia Ritter, Paul G. Layer, Christiane Thielemann

**Affiliations:** 1 University of Applied Sciences Aschaffenburg, Wuerzburger Strasse 45, D-63743 Aschaffenburg, Germany; 2 Technische Universität Darmstadt, Germany; 3 GSI, Darmstadt, Germany

**Keywords:** space radiation, heavy ion, IBER, cardiomyocytes, microelectrode arrays

## Abstract

Since essentially no information is available on the effects of high linear energy transfer (LET) radiation on the heart, an assessment of possible late effects on the cardiovascular system is important with respect to the planning of manned long-term space missions [ 1, 2]. In order to examine if and to what extent heart muscle cells are affected by an exposure to heavy ions, primary avian cardiomyocytes were isolated, cultured *in vitro* and exposed at GSI (Darmstadt) to different ion species (carbon, titanium and nickel). Investigation of electrophysiologic radiation effects was performed on cells grown on a microelectrode array (MEA) allowing the monitoring of beat rate, spike shape, field action potential duration and signal conduction pathways across the electrode array. Data were analysed using the DrCell software [ 3]. In parallel, immunohistological stainings were conducted to examine cell cycle progression, DNA-damage repair and apoptosis.

Our preliminary data indicate that primary cardiac cells possess a high robustness toward ionizing radiation as cultures receiving doses of up to 7 Gy still show unaltered electrophysiologic activity. This is exemplarily shown in Fig. 1, where the beat rate of cells irradiated with 2 and 7 Gy carbon ions has been plotted. Figure 1 also illustrates that within each treatment group, inter-sample variations are quite large and thus do not allow detection of subtle changes in cellular electrophysiology, probably due to the embryonic character of the cells.

Interestingly, in terms of the formation of double-strand breaks (DSBs) and cell cycle progression delay, the cultures showed a dose-dependent reduction in proliferation and DSB accumulation.

Taken together, our electrophysiologic studies indicate that the large variations within the same treatment group of avian embryonic cardiomyocytes obscure the detection of radiation effects. To account for this problem, we will use in further studies human cardiomyocytes differentiated from iPS cells, as these cells resemble more closely the condition of an adult human heart and do not undergo developmental changes as embryonic cell cultures do.

**Fig. 1.**
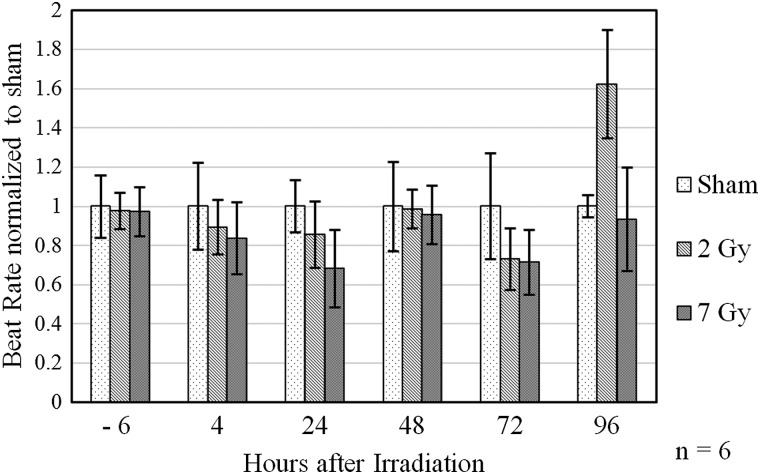
Normalized beat rates measured in samples before and after exposure to carbon ions. (spread-out Bragg peak, LET: 75 keV/µm). Average beat rates range from 22 to 53 beats/min.

Normalized beat rates measured in samples before and after exposure to carbon ions. (spread-out Bragg peak, LET: 75 keV/µm). Average beat rates range from 22 to 53 beats/min.
